# Networked dynamic systems with higher-order interactions: stability versus complexity

**DOI:** 10.1093/nsr/nwae103

**Published:** 2024-03-18

**Authors:** Ye Wang, Aming Li, Long Wang

**Affiliations:** Center for Systems and Control, College of Engineering, Peking University, Beijing 100871, China; Center for Systems and Control, College of Engineering, Peking University, Beijing 100871, China; Center for Multi-Agent Research, Institute for Artificial Intelligence, Peking University, Beijing 100871, China; Center for Systems and Control, College of Engineering, Peking University, Beijing 100871, China; Center for Multi-Agent Research, Institute for Artificial Intelligence, Peking University, Beijing 100871, China

**Keywords:** networked system, set structure, higher-order interaction, stability criteria

## Abstract

The stability of complex systems is profoundly affected by underlying structures, which are often modeled as networks where nodes indicate system components and edges indicate pairwise interactions between nodes. However, such networks cannot encode the overall complexity of networked systems with higher-order interactions among more than two nodes. Set structures provide a natural description of pairwise and higher-order interactions where nodes are grouped into multiple sets based on their shared traits. Here we derive the stability criteria for networked systems with higher-order interactions by employing set structures. In particular, we provide a simple rule showing that the higher-order interactions play a double-sided role in community stability—networked systems with set structures are stabilized if the expected number of common sets for any two nodes is less than one. Moreover, although previous knowledge suggests that more interactions (i.e. complexity) destabilize networked systems, we report that, with higher-order interactions, networked systems can be stabilized by forming more local sets. Our findings are robust with respect to degree heterogeneous structures, diverse equilibrium states and interaction types.

## Teaser

This study establishes stability criteria for networked dynamic systems with higher-order interactions by employing set structures to capture empirical higher-order interactions, and obtains a simple rule to stabilize such systems.

## INTRODUCTION

Explaining the intricate effect of community structures on the stability of complex systems has been a long-lasting challenge in mathematics, control and ecology [1–11]. Stability, one of the most important indicators in complex systems, portrays the ability of a system to return to its equilibrium after external perturbations [12–17]. By analyzing the eigenvalue of the linearized community matrix, May [1,2] first proved that, for a random community where species interact randomly, achieving stability becomes increasingly challenging as community diversity and complexity rise. Following May’s framework, both theoretical analysis and experiments have shown that the underlying structure can significantly affect the stability of complex systems, such as heterogeneous, multi-layer, temporal and higher-order structures [18–22].

Previous studies generally use complex networks to model various community structures, where nodes represent species and edges represent pairwise interactions [23–29]. Despite the many deep insights gained about the stability of complex systems under different structures, little attention has been paid to higher-order interactions with more than two species, namely, the interaction between two species might be affected by other species [30,31]. The hypergraph formalism is a comprehensive framework for studying higher-order interactions, which is fundamentally equivalent to the set structure [32]. Indeed, the set structure represents a typical community structure that incorporates both pairwise and higher-order interactions. According to ‘evolutionary set theory’ [33–35], species in the realistic system are typically distributed over sets, where species within the same set interact with each other, and each species may belong to multiple sets based on multiple criteria. For example, goldfish and lotus plants in the same pond could be considered members of the same set based on their shared location, but they would be classified into distinct sets based on their taxonomic differences. By grouping species into multiple sets, set structures offer a more comprehensive perspective on species relationships within a networked system. Indeed, previous studies have demonstrated that the set structure has a significant impact on the evolutionary dynamics of networked systems [33–35].

Nonetheless, compared with other extensively studied network structures [30,36–38], the effect of the set structure on the stability of complex systems has been poorly understood. Prior explorations have demonstrated that different network structures, such as heterogeneous and modularity structures [39,40], could lead to various stability criteria even under the same interaction type. Based on the framework of ‘evolutionary set theory’ and stability theory [12,33–35,41,42], here we investigate the impact of the set structure on the stability of complex systems under various interaction types, and analyze the corresponding stability criteria in set structured systems. To further understand the role of set structures and higher-order interactions, we also compare the stability of set structured systems with that of corresponding unstructured (set-free) systems [4,18]. Our results indicate that the number of sets each species belongs to ($\mathcal {K}$) and the overall number of sets ($\mathcal {G}$) determine the stability of set structured systems. Surprisingly, we find that the destabilizing effect of increasing connectivity revealed by previous studies [1,43] can be counteracted in set structured systems by reducing the expected number of common sets for two randomly selected species (i.e. order, $\mathcal {H}=\mathcal {K}^2/\mathcal {G}$). Furthermore, we provide a simple rule, $\mathcal {H}< 1$, to enhance stability in set structured systems, which is suitable in various realistic scenarios such as heterogeneous structures, diverse equilibrium abundances and diverse interaction types.

## RESULTS

### Model

Here we employ the classical ordinary differential equations to describe the dynamics of species interactions [4,18,43]


(1) \begin{eqnarray*}
\frac{{\rm d} {\boldsymbol {X}}(t)}{{\rm d} t}={\rm diag}({\boldsymbol{ X}}(t)){\boldsymbol {f}}({\boldsymbol{X}}(t)),
\end{eqnarray*}

where ${\boldsymbol X}(t)=[X_{1}(t),X_{2}(t), \dots , X_{S}(t)]^\top$ contains the abundance (state) of each species at time *t* and ${{\boldsymbol f}}({\boldsymbol X}(t))$ captures the specific interactions among species. The diagonal matrix ${\rm diag}({\boldsymbol X}(t))$ has the elements of ${\boldsymbol X}(t)$ on its diagonal and zeros elsewhere. We call ${\boldsymbol X}^{*}=[X_{1}^{*}, X_{2}^{*}, \dots , X_{S}^{*}]^\top$ a feasible equilibrium point satisfying ${\boldsymbol f}({\boldsymbol X}^{*})=\bf{0}$ and $X_{i}^{*}> 0,\, i=1,2,\dots , S$.

Following the framework of stability theory, we linearize the networked system to assess the stability of the system near ${\boldsymbol X}^{*}$, which subsequently gives


(2) \begin{eqnarray*}
\frac{\mathrm{d} {\boldsymbol x}(t)}{\mathrm{d} t} = \mathrm{diag}({\boldsymbol X}^{*}) {{\boldsymbol J}} {{\boldsymbol x}}(\it t)={\boldsymbol M}{\boldsymbol x}(\it t)
\end{eqnarray*}

with ${\boldsymbol J}={\partial {\boldsymbol f}}/{\partial {\boldsymbol X}}$ and ${\boldsymbol x}(t)={{\boldsymbol X}}(t)-{\boldsymbol X}^{*}$. Here, $\mathrm{diag}({\boldsymbol X}^{*}) {{\boldsymbol J}}$ is the community matrix of the system, which is often denoted by ${{\boldsymbol M}}$, whose element *M_ij_* describes the effect that species *j* has on species *i*, and *M_ii_* is the intrinsic effect of species *i*. Mathematically, the stability of the linearized system near the equilibrium is determined by the largest real part among all eigenvalues of ${\boldsymbol M}$, Re($\lambda _{{\boldsymbol M},1}$). Specifically, a negative Re($\lambda _{{\boldsymbol M},1}$) indicates an asymptotically stable system near the equilibrium, while the equilibrium is unstable if Re($\lambda _{{\boldsymbol M},1}$) is positive.

In our model, we consider the effect of set structures on the stability of networked systems, where nodes represent species and nodes within the same set can interact with each other. Let $\mathcal {G}$ denote the number of sets and each node belong to $\mathcal {K}$ randomly chosen sets ($\mathcal {K}\le \mathcal {G}$); we then construct the community matrix ${\boldsymbol M}$ as follows:

- for any species *i* and set *g, g_i_* = 1 if *i* belongs to *g* and *g_i_* = 0 otherwise;
- for any set *g*, we define $(M_{ij}^{g},M_{ji}^{g})$ as the interaction strength between species *i* and *j* in set *g*;
- we draw $(M_{ij}^{g},M_{ji}^{g})$ from a bivariate distribution with probability *C* if both species *i* and *j* are in set *g* (*g_i_g_j_* = 1), and we set $M_{ij}^{g}=M_{ji}^{g}=0$ otherwise;
- for the community matrix ${\boldsymbol M}$, $M_{ij}=\sum ^{\mathcal {G}}_{g=1}M_{ij}^{g}$;
- for diagonal elements *M_ii_*, we set *M_ii_* = −*d*, where *d* is a positive constant.

**Figure 1.**
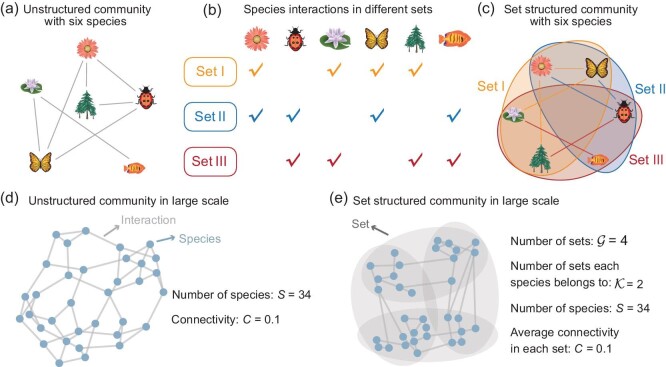
Construction of the unstructured and set structured systems. (a) Interactions between six species in the unstructured system. Each species interacts with others in the random network, where gray lines depict the interaction between each pair of species. (b) The distribution of six species over three sets. Each species belongs to two sets randomly among the three sets, and the expected number of species in each set is four. (c) Interactions among six species in the set structured system. Each species interacts with others within the same set with probability *C*, where colored lines correspond to specific interactions in each set. (d) A large-scale unstructured (random) system. Each species is represented by a node, and edges represent interactions between species. Here the number of species *S* = 34 and the connectivity *C* = 0.1. (e) A large-scale set structured system. Each species (node) belongs to two sets among four sets. Species within the same set interact with probability *C* (edges). Except for the setting of sets, other parameters in the set structured system are identical to those in the unstructured system (number of species, connectivity, etc.).

In classical studies on the stability of complex systems, the unstructured model supposes that each pair of species interacts with probability *C* (i.e. connectivity). The corresponding community matrix $\hat{{\boldsymbol M}}$ of the unstructured system primarily indicates pairwise interactions [8,38] (Fig. 1a and d). These assumptions facilitate the theoretical analysis of complex systems by focusing predominantly on pairwise interactions within networked systems, which can be denoted by edges. However, unstructured models cannot comprehensively capture the dynamic nature of real-world systems, where species interactions are not exclusively pairwise. In reality, species are allowed to interact multiple times since they may belong to various common sets in the set structured system, whose community matrix ${\boldsymbol M}=\sum ^{\mathcal {G}}_{g=1}{\boldsymbol M}^{g}$ incorporates both pairwise and higher-order interactions (Fig. 1b, c and e). Indeed, the number of sets that any two species have in common is defined as the order between the two species. The expected number of common sets for two randomly chosen species ($\mathcal {H}$) is the overall order of the set structured system. Different from the classical networked systems [37,44], here we explore the stability of set structured systems and reveal the effect of the combination of pairwise and higher-order species interactions on community stability. For interactions between species, we mainly consider the following four types [4,18] (see Section S1 for details).

- Random: interaction strengths between two species are drawn from a normal distribution *N*(0, σ^2^) independently.
- Exploitative (+/−): interaction strengths between two species have opposite signs. One is drawn from the distribution −|*N*(0, σ^2^)|, and the other is drawn independently from the distribution |*N*(0, σ^2^)|.
- Mutualistic (+/+): interaction strengths between two species have positive signs and are drawn from |*N*(0, σ^2^)| independently.
- Competitive (−/−): interaction strengths between two species have negative signs and are drawn from −|*N*(0, σ^2^)| independently.

These interaction types can effectively describe complex real-world interaction scenarios. For example, when two species compete for limited resources, their interactions are competitive. Conversely, when two species cooperate for mutual benefit, their interactions are mutualistic.

### Stability criteria for diverse interaction types

In contrast to classical unstructured (set-free) systems where species interact with probability *C* [43,45], here we study the stability of set structured systems and investigate the effect of sets on community stability. We compare the largest real part among all eigenvalues of ${\boldsymbol M}$ and $\hat{{\boldsymbol M}}$, which is the community matrix of the corresponding unstructured (set-free) systems. To ensure a fair comparison, the parameters of both set structured and unstructured systems are set to be identical, such as the number of species (*S*), the connectivity (*C*) and the interaction types. Theoretically, we provide the stability criteria to estimate the stability of set structured systems. Since a negative Re($\lambda _{{\boldsymbol M},1}$) indicates a stable system, we use −Re($\lambda _{{\boldsymbol M},1}$) and −Re($\lambda _{\bf{\hat{M}},1}$) to represent the stability of set structured and unstructured systems, respectively [18,46].

**Figure 2.**
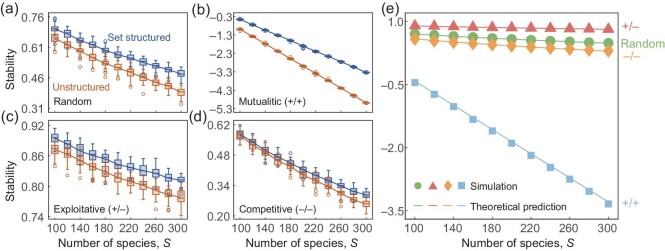
Set structures improve community stability. (a–d) The stability of set structured and corresponding unstructured systems with an increasing number of species, *S*. Specific interaction types in each panel correspond to random, mutualistic (+/+), exploitative (+/−) and competitive (−/−), respectively. (e) Theoretical predictions (lines) and numerical simulations (dots) on the stability of set structured systems under different interaction types. We set the number of sets $\mathcal {G}=50$, the number of sets each species belongs to $\mathcal {K}=6$, the connectivity *C* = 0.5 and σ = 0.05, and we vary the number of species *S* from 100 to 300. Each simulation result (box chart and dot) is obtained from over 50 replicates.

We find that, compared with the unstructured system, the set structure has a profound effect on stabilizing the networked system if the number of sets is much larger than the number of sets that each species belongs to (Fig. 2a–d), regardless of the specific interaction types (random, exploitative, mutualistic and competitive).

Next, we give the corresponding stability criterion for different interaction types in set structured systems in Table 1 [4,47–49] (see the Methods section and Section S2 for details). We verify our theoretical analysis of the stability criteria in set structured systems through Monte Carlo simulations, and our theoretical predictions are in good agreement with the numerical results (Fig. 2e and Fig. S1).

**Table 1.** Stability criteria for different interaction types in set structured and unstructured systems.

Interaction type	Stability criterion (set structured community)	Stability criterion (unstructured community)
Random	$\sqrt{SC{\mathcal {K}^2}/{\mathcal {G}}}< {d}/{\sigma }$	$\sqrt{SC}< {d}/{\sigma }$
Mutualistic	$(S-1)C({\mathcal {K}^2}/{\mathcal {G}}) \sqrt{2/\pi }< {d}/{\sigma }$	$(S-1)C \sqrt{2/\pi }< {d}/{\sigma }$
Exploitative	$\sqrt{SC({\mathcal {K}^2}/{\mathcal {G}}})(1-2/\pi )< {d}/{\sigma }$	$\sqrt{SC}(1-2/\pi )< {d}/{\sigma }$
Competitive	$F(C{\mathcal {K}^2}/{\mathcal {G}})+C({\mathcal {K}^2}/{\mathcal {G}}) \sqrt{2/\pi }< {d}/{\sigma }$	$F(C)+C\sqrt{2/\pi }< {d}/{\sigma }$

### General rule to stabilize complex communities

Furthermore, we give a quantitative condition under which stability is enhanced in set structured systems, compared with that in unstructured systems. The expected number of common sets, $\mathcal {H}$, for two randomly chosen distinct species is (see Section S2.1 for details)


(3) \begin{eqnarray*}
\mathcal {H}=\frac{\mathcal {K}^2}{\mathcal {G}}.
\end{eqnarray*}

Considering that species within the same set interact with probability *C*, the expected interaction times, *T*, for any two species is thus $T=C\mathcal {H}=C{\mathcal {K}^2}/{\mathcal {G}}.$ According to Table 1, we find that *T* is a variable that describes ‘complexity’ in set structured systems, corresponding to the connectivity *C* in unstructured systems. Intuitively, increasing connectivity *C* decreases the stability of networked systems since it increases the complexity of the system [1,2]. Following May’s seminal framework, species interact more frequently with increasing *T* in set structured systems, making the system more complex. Our results show that the stability of set structured systems decreases with increasing *T*, which increases the complexity (the expected interaction times for any two species) of the whole system.

Surprisingly, we find that, the stability is negatively correlated with the expected number of common sets $\mathcal {H}$ in set structured systems for random, exploitative or mutualistic interactions. Compared with the corresponding unstructured systems with the same coefficients, the set structured systems are more stable (i.e. $-{\rm Re}(\lambda _{\bf{\hat{M}},1})< -{\rm Re}(\lambda _{{\bf{M}},1}$)) if $\mathcal {H}< 1$, namely,


(4) \begin{eqnarray*}
\frac{\mathcal {K}^2}{\mathcal {G}}< 1.
\end{eqnarray*}

This means that in set structured systems species may form more local sets to overcome the destabilization effect of the increasing number of interactions among species. Specifically, we provide a critical mathematical condition to enhance stability in set structured systems, which is $\mathcal {K}^2< \mathcal {G}$.

**Figure 3.**
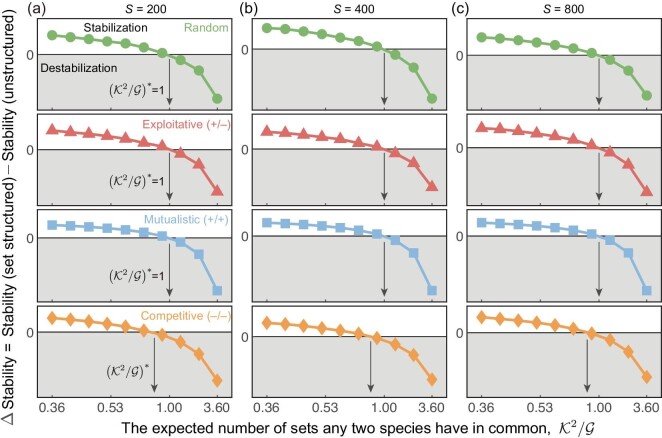
Confirmation of the simple rule, $\mathcal{K}^2/\mathcal {G}< 1$, to enhance stability in set structured systems. (a–c) The ΔStability of set structured and corresponding unstructured systems with increasing $\mathcal {K}^2/\mathcal {G}$ in small (*S* = 200), medium (*S* = 400) and large size (*S* = 800) communities, respectively, where ΔStability = Stability(setstructured) − Stability(unstructured). Interaction types are random, exploitative (+/−), mutualistic (+/+) and competitive (−/−) (in order from top to bottom). We highlight the prediction of the critical $(\mathcal {K}^2/\mathcal {G})^{*}$ under different interaction types. The simple rule accurately predicts the critical $(\mathcal {K}^2/\mathcal {G})^{*}=1$ in random, exploitative and mutualistic communities. The results suggest that $\mathcal {K}^2/\mathcal {G}< 1$ is necessary but not sufficient in the competitive community, as the critical $(\mathcal {K}^2/\mathcal {G})^{*}< 1$ when the interaction type is competitive. For set structured systems, we set the number of sets each species belongs to $\mathcal {K}=6$, the connectivity *C* = 0.5 and σ = 0.05, and we vary the number of sets $\mathcal {G}$ from 10 to 100. For unstructured systems, we employ the same parameters (*C*, σ) as those in set structured cases. Each result is obtained from over 50 replicates.

Our theoretical predictions are consistent with Monte Carlo simulations when the interaction types are random, exploitative or mutualistic. We find that, when the interaction type in set structured systems is competitive, the critical condition is more strict, and only a larger $\mathcal {G}$ can stabilize the set structured system since the stability criterion is more sensitive to the varying $\mathcal {G}$ in competitive cases (Fig. 3a–c). Our findings indicate that higher-order species interactions ($\mathcal {H}$) have a double-sided role in community stability, and the stability is enhanced only if $\mathcal {H}< 1$. These results are also applicable in more general cases, such as mixed interaction types (Fig. S5) and various community connectivity (Fig. S2).

**Figure 4.**
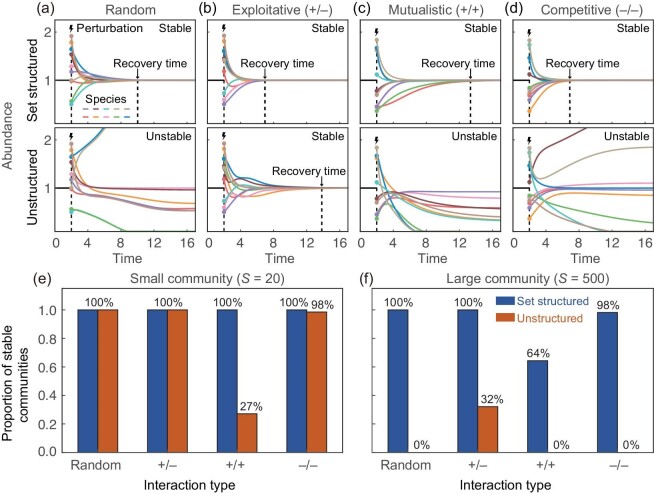
Simulated data verifies the stabilizing effect of set structures on community stability. (a–d) Simulation results illustrate the evolution of species abundance over time in set structured and corresponding unstructured systems with the same perturbations. We find that set structures exhibit a significant stabilizing effect on community stability if $\mathcal {K}^2/\mathcal {G}< 1$, irrespective of specific interaction types, such as random, exploitative (+/ −), competitive (−/ −) and mutualistic (+/ +) interactions (in order from left to right). Even though both set structured and unstructured systems with exploitative (+/ −) interactions return to equilibrium after perturbations, set structured systems show a notably shorter recovery time. Parameters are $S=10,\sigma =0.5,C=0.25,\mathcal {G}=6,\mathcal {K}=2$. (e, f) The proportion of stable communities among 1000 simulated set structured and corresponding unstructured communities. In small communities (*S* = 20), both set structured and unstructured systems tend to maintain stability. However, in large communities (*S* = 500), set structured systems sustain stability, while their unstructured counterparts grapple with instability. Parameters are $\sigma =0.25,C=0.25,\mathcal {G}=200,\mathcal {K}=2$.

To showcase the usefulness of our method and to provide quantitative comparisons with existing work [4], we next generate a significant amount of simulated data to validate the stabilizing effect of set structures on community stability (see Section S3 for details). Our simulations track the dynamical change of species abundance in both set structured and unstructured systems following identical external perturbations. These numerical experiments reveal that if the theoretical condition $\mathcal {K}^2/\mathcal {G}< 1$ is satisfied, the relative abundance of each species converges more rapidly in set structured systems than in unstructured systems, even when both systems are stable. Furthermore, the set structured system can still be stable when the corresponding unstructured systems are unstable for $\mathcal {K}^2/\mathcal {G}< 1$ (Fig. 4a–d). In addition, we also calculate the proportion of stable communities among numerous simulated communities and show that set structured systems are more likely to be stable in large complex communities if $\mathcal {G} \gg \mathcal {K}^2$ (Fig. 4e and f).

### Effect of degree heterogeneity on community stability

**Figure 5.**
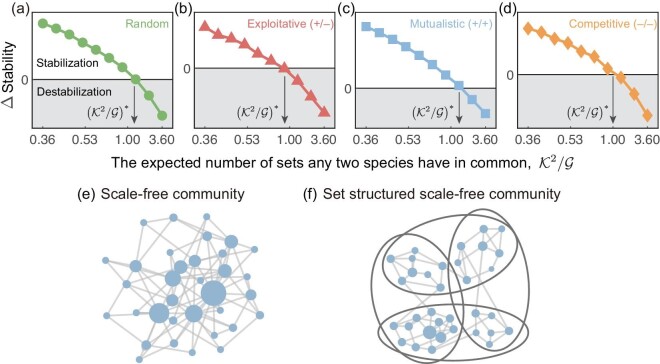
Confirmation of the simple rule, $\mathcal {K}^2/\mathcal {G}< 1$, in heterogeneous systems. (a–d) The ΔStability of set structured and corresponding unstructured systems with increasing $\mathcal {K}^2/\mathcal {G}$ in degree heterogeneous communities under random, exploitative (+/−), mutualistic ( + / + ) and competitive (−/−) interaction types (in order from left to right). We highlight the critical $({\mathcal {K}^2}/{\mathcal {G}})^{*}\approx 1$ in degree heterogeneous communities. (e) Degree heterogeneous (scale-free) system. Each species is represented by a node, and edges represent interactions between species. Here the number of species *S* = 34 and the average connectivity *C* = 0.2. (f) Set structured degree heterogeneous (scale-free) system. Each species (node) belongs to two sets among four sets, and other corresponding parameters are identical to those in the unstructured system (number of species, average connectivity, etc.). In (a–d), we set the connectivity *C* = 0.2, with the other parameters the same as those in Fig. 3a. For unstructured systems, we employ the same parameters (*C*, σ) as those in set structured cases. Each result is obtained from over 50 replicates.

We have investigated the effect of set structures on community stability where species within the same set interact randomly. However, related studies have shown that the topological structures in empirical systems can also be non-random and exhibit degree heterogeneity [50,51], indicating that species’ degree distributions vary across different levels of generality. Specifically, degree heterogeneity refers to the uneven distribution of connections of all nodes. This concept signifies that, within a network, some nodes possess a high degree of connectivity, whereas others exhibit a low degree. For example, in transportation networks like airline routes, certain airports serve as major hubs with numerous direct flights to various destinations, while smaller airports have fewer connections. Next, we demonstrate that our framework may analyze degree heterogeneous networks (see the Methods section for details) and investigate the effect of degree heterogeneity on community stability of set structured systems (Fig. 5e and f).

Remarkably, our conclusions still hold in degree heterogeneous networks, indicating that set structures play a dominant role in community stability. Although degree heterogeneity can lead to fluctuating results, we show that the critical $({\mathcal {K}^2}/{\mathcal {G}})^{*}$ is consistently around 1 (Fig. 5a–d), irrespective of specific interaction types (random, exploitative, mutualistic and competitive). Moreover, our results are robust for mixed interaction types (Fig. S6).

Following May’s framework, related studies show that increasing connectivity (‘complexity’) decreases stability in networked systems, regardless of the degree distribution of the system [4]. However, our results reveal that increasing the number of sets $\mathcal {G}$ overcomes the destabilization effect of increasing connectivity in set structured systems in both degree homogeneity and heterogeneity networks. And the general rule $({\mathcal {K}^2}/{\mathcal {G}}) < ({\mathcal {K}^2}/{\mathcal {G}})^{*}\approx 1$ is a critical condition to enhance stability in set structured systems compared with unstructured systems.

### Model extension

After exploring the effect of degree heterogeneity on the stability of set structured and unstructured systems, we extend our models to more complex scenarios, including local symmetric structures and diverse equilibrium abundances [52,53], to verify the robustness of our findings.

**Figure 6.**
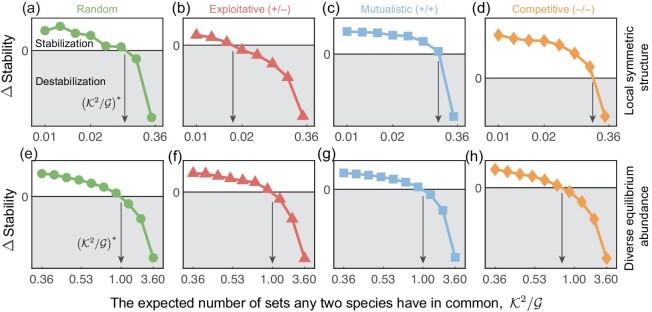
Forming more local sets can improve the stability in local symmetric structures and with diverse equilibrium abundances. The ΔStability of set structured and corresponding unstructured systems with increasing $\mathcal {K}^2/\mathcal {G}$ in local symmetric communities (a–d) and with diverse equilibrium abundances in homogeneous communities (e–h) under random, exploitative (+/−), mutualistic ( + / + ) and competitive (−/−) interaction types (in order from left to right). We highlight the critical $({\mathcal {K}^2}/{\mathcal {G}})^{*}\approx 1$ among all panels. (a–d) The average degree in both set structured and unstructured systems *k* = 4. (e–h) The equilibrium abundance is drawn uniformly from the interval [0.75,1.25]. We set parameters identical to those in Fig. 3a, and vary the number of sets $\mathcal {G}$ from 100 to 1000 (a–d) or 10 to 100 (e–h). Each result is obtained from over 50 replicates.

Here we investigate the effect of local symmetric structures with heterogeneous degree distributions on the stability of set structured systems. Specifically, the average degree in unstructured systems is equivalent to that in each set in set structured systems (see the Methods section for details). Furthermore, in local symmetric structures, the complexity of set structured systems is higher than that of unstructured systems, as the overall average degree is higher than the average degree in each set in set structured systems. Surprisingly, despite the higher complexity of set structured systems in local symmetric structures, set structured systems can still be more stable than unstructured systems if $\mathcal {G} \gg \mathcal {K}^2$. Under different interactions (random, exploitative, mutualistic and competitive), the critical $({\mathcal {K}^2}/{\mathcal {G}})^{*}$ varies in local symmetric structures (Fig. 6a–d).

Moreover, we explore the effect of equilibrium abundance on the stability of set structured and unstructured systems. In realistic systems, species may have different equilibrium abundances, and previous studies have shown that these differences can significantly affect community stability. In previous sections, we postulated that the equilibrium abundance ${X_i}^{*}=1,\, i=1,2,\dots ,S$. Here we extend this assumption by allowing for diverse equilibrium abundances and investigate their effect on the stability of set structured and unstructured systems in both homogeneous and heterogeneous communities. Our findings reveal that the critical $({\mathcal {K}^2}/{\mathcal {G}})^{*}$ remains close to 1, regardless of the diverse equilibrium abundances (Fig. 6e–h). We also examine different equilibrium abundance distributions, such as log-normal and half-normal distributions, and find that they do not qualitatively alter our results (Fig. S3). We further consider degree heterogeneous communities and demonstrate that the critical $({\mathcal {K}^2}/{\mathcal {G}})^{*}$ is still near 1 under diverse equilibrium abundances in such communities (Figs. S4, S7–S9).

## DISCUSSION

Higher-order interactions are of paramount importance in various fields such as network science, ecology, mathematics and sociology [30–32]. In particular, extensive research has been devoted to understanding the impact of higher-order interactions on complex systems by considering third- or fourth-order interactions [30,31]. Our study introduces set structures, equivalent to hypergraphs [32], as a novel framework for analyzing higher-order species interactions. Within this framework, species are organized into distinct sets and interact with other species within the same set, potentially encompassing interactions of any order within the community. This general and innovative approach promises to greatly enhance our comprehension of intricate community dynamics. By incorporating both pairwise and higher-order interactions, set structures effectively capture the complexity of interactions in networked systems.

Indeed, as a kind of hypergraph [32], set structures are ubiquitous in both nature and human society, from microorganisms and animals to human populations [33,34]. Specifically, species may belong to multiple sets simultaneously under different classification criteria, playing different roles and functions in each set. For example, whales are both marine and mammalian in the sea system, while sharks are marine but not mammalian. Previous studies demonstrate that set structures quantitively influence the evolutionary outcomes of complex systems [34,35]. Here we consider complex systems with higher-order interactions by introducing set structures, and investigate the stability of set structured systems. We establish the stability criteria in set structured systems. Our findings demonstrate that, contrary to the classical ‘diversity-stability debate’ [1,2], increasing the number of sets, $\mathcal {G}$, has a stabilizing effect on set structured systems, which can overcome the destabilization effect of increasing connectivity.

Surprisingly, we find that, compared with the corresponding unstructured systems, there exists a simple general rule to stabilize set structured systems. Moreover, we have theoretically derived that the set structured system is more stable when $({\mathcal {K}^2}/{\mathcal {G}})< ({\mathcal {K}^2}/{\mathcal {G}})^{*}\approx 1$, irrespective of interaction types. Furthermore, we find that set structures play a significant role in community stability, and the effect of set structures on system stability is robust under various parameters, including the number of species, connectivity, etc. Remarkably, we also discuss the effect of degree heterogeneous structures, diverse equilibrium abundances and mixed interaction types on the stability of set structured systems. Our rule still holds when considering these realistic factors. Overall, our study provides a new perspective on understanding the role of set structures in community stability and offers a general framework to study the role of set structures in complex systems.

Our model employs set structures as a framework to investigate the impact of higher-order interactions on the stability of networked dynamic systems. It is worth noting that there exist various ways to characterize higher-order interactions, such as hyperedges and simplicial complexes. Therefore, the exploration of multiple definitions of higher-order interactions holds the potential for a comprehensive examination of their impact on the stability and complexity of networked systems. Additionally, the stability of complex systems in the real world is subject to various factors such as stochasticity, nonlinearity and reactivity [46,54–57]. Future work based on our framework can further consider these factors, contributing to a more thorough understanding of complex systems. Furthermore, exploring real-world networks with set structures and diverse species interactions represents a promising direction for future research based on our theoretical findings, with the key challenge being the acquisition of relevant data. Therefore, efforts to obtain and analyze such data can greatly enhance our understanding of the stability of empirical systems. In addition, previous work has pointed out that environmental feedback plays a pivotal role in shaping the dynamics of complex systems [56]. Combining environmental feedback with higher-order interactions can extend our work into the broader context of co-evolutionary dynamics research, deepening our understanding of the impact of higher-order interactions on the stability of networked dynamic systems.

Another potential extension points to the future application of our model in more realistic scenarios, such as multi-layer and time-delay systems [18,46]. By investigating the role of higher-order interactions in these realistic complex systems, we can better understand the system stability. This exploration also highlights that higher-order interactions impact not only the stability and complexity of complex systems, but also their fundamental functions. For example, by identifying the sets most critical to community stability, we can prioritize conservation efforts and develop targeted interventions to protect those sets. These extensions will help us understand more about the system stability and have profound theoretical and practical implications, which also significantly enrich the investigation of the effect of set structures on the stability of complex systems.

## METHODS

### Heterogeneous community matrix

In each set $g\ (g=1,2,\dots , \mathcal {G})$ we construct a scale-free network ${\boldsymbol G}^{g}$ with connectivity *C*, where nodes represent species and edges capture interactions. The construction process of scale-free networks typically follows the preferential attachment mechanism [50], where new nodes are more likely to connect to existing nodes with high degrees. If species *i* and *j* are in the same set *g* (*g_i_* = *g_j_* = 1) and there exists an edge between them, we sample $(M_{ij}^{g},M_{ji}^{g})$ from a given bivariate distribution; otherwise, $M_{ij}^{g}=M_{ji}^{g}=0$. Different interaction types correspond to distinct bivariate distributions. We have


(5) \begin{eqnarray*}
M_{ij}=\sum ^{\mathcal {G}}_{g=1}M_{ij}^{g}.
\end{eqnarray*}

In the corresponding heterogeneous unstructured systems, we first construct a scale-free network ${\boldsymbol{G}}$ with connectivity *C*. For any species *i* and *j*, we sample $(\hat{M}_{ij},\hat{M}_{ji})$ from the same distribution as that in heterogeneous set structured systems if *G_ij_* = *G_ji_* = 1; otherwise, $\hat{M}_{ij}=\hat{M}_{ji}=0$.

### Local symmetric structure community matrix

In each set *g*, we construct a scale-free network ${\boldsymbol G}^{g}$ whose average degree is *k*. If *g_i_g_j_* = 1 and there exists an edge between species *i* and *j*, we sample $(M_{ij}^{g},M_{ji}^{g})$ from a given bivariate distribution; otherwise, $M_{ij}^{g}=M_{ji}^{g}=0$. Then, we have $M_{ij}=\sum ^{\mathcal {G}}_{g=1}M_{ij}^{g}$.

In the corresponding unstructured systems, we also construct a scale-free network ${\boldsymbol{G}}$ whose average degree is *k*. We sample $(\hat{M}_{ij},\hat{M}_{ji})$ from the same distribution as that in set structured systems if *G_ij_* = *G_ji_* = 1; otherwise, $\hat{M}_{ij}=\hat{M}_{ji}=0$.

### Stability criteria

(1) *Random*. When interactions between species are random, for the community matrix ${\boldsymbol M}$, we have $\mathbb {E}(M_{ij})_{i \ne j}=0$ and $\text{Var}(M_{ij})_{i \ne j}=C{\mathcal {K}^2}\sigma ^2 /{\mathcal {G}}$, where $\mathcal {G}$ is the number of sets and $\mathcal {K}$ is the number of sets each species belongs to. The above relations give the corresponding stability criterion for random interactions as shown in Table 1 (see Section S2.2).

(2) *Exploitative* (+/−). For the exploitative case that interactions between species in each set are exploitative, we have $\mathbb {E}(M_{ij})_{i \ne j}=0$, $\mathbb {E}(M_{ij}M_{ji})_{i \ne j}=-C({\mathcal {K}^2}/{\mathcal {G}} ) \mathbb {E}^2(|X|)$ and $\text{Var}(M_{ij})_{i \ne j}=C({\mathcal {K}^2}/{\mathcal {G}})\text{Var}(X)$, where *X* ∼ *N*(0, σ^2^). The above relations give the corresponding stability criterion for exploitative interactions as shown in Table 1 (see Section S2.3).

(3) *Mutualistic* ( + / + ). In the mutualistic case, $(M_{ij}^{g},M_{ji}^{g})$ are drawn from the distribution |*X*| with probability *C* if species *i* and *j* both belong to set *h*. For the community matrix ${\boldsymbol M}$, we have $\mathbb {E}(M_{ij})_{i \ne j}=C({\mathcal {K}^2}/{\mathcal {G}}) \mathbb {E}(|X|)$ and $\text{Var}(M_{ij})_{i \ne j}=C({\mathcal {K}^2}/{\mathcal {G}})\mathbb {E}(X^2)- (C[{\mathcal {K}^2}/{\mathcal {G}}]\mathbb {E}(|X|))^2$, where *X* ∼ *N*(0, σ^2^). The above relations give the corresponding stability criterion for mutualistic interactions as shown in Table 1 (see Section S2.4).

(4) *Competitive* (−/−). We finally discuss the competitive case where species interact competitively. Similarly, we have $\mathbb {E}(M_{ij})_{i \ne j}=-C({\mathcal {K}^2}/{\mathcal {G}}) \mathbb {E}(|X|)$ and $\mathbb {E}(M_{ij}M_{ji})_{i \ne j}=C({\mathcal {K}^2}/{\mathcal {G}}) \mathbb {E}^2(|X|)$. The above relations give the corresponding stability criterion for competitive interactions as shown in Table 1 (see Section S2.5), where *F*(*x*) satisfies


(6) \begin{eqnarray*}
F(x)=\sqrt{Sx\bigg (1-2\frac{x}{\pi }\bigg )}\bigg (1+\frac{2(1-x)}{\pi -2x}\bigg ).
\end{eqnarray*}

Note that our stability criteria for competitive interactions in set structured systems are obtained through the approximate estimation, which is violated under the condition that $C{\mathcal {K}^2}/{\mathcal {G}} \ll 1$.

## Supplementary Material

nwae103_Supplemental_File

## Data Availability

All data analyzed can be reproduced using the codes available online at https://github.com/yewang0109/NetStaCom. The codes were written in MathWorks Matlab R2022b.
