# Supplementary material for: Networked dynamic systems with higher-order interactions: stability versus complexity
Source: Natl Sci Rev. 2024 Mar 18;11(9):nwae103. doi: 10.1093/nsr/nwae103 (PMC11321256; doi:10.1093/nsr/nwae103)
Supplement: nwae103_Supplemental_File [file nwae103_supplemental_file.pdf]

Supplementary Information for

Networked dynamic systems with higher-order interactions: stability versus complexity

Ye Wang, Aming Li & Long Wang

1 Constructing community matrices

As shown in the main text, we focus on the largest real part among all eigenvalues of the community matrix, $\boldsymbol{M}$. Specific interaction types in set structured systems determine the corresponding community matrices. Here we consider four interaction types (random, exploitative, mutualistic, and competitive) and show how to construct corresponding community matrices in set structured systems where species within the same set interact with probability C. This construction process can also be extended to heterogeneous and local symmetric structure communities. Our main parameters are: number of species, S, number of sets, $\mathcal{G}$, number of sets each species belongs to, $\mathcal{K}$, connectivity in each set, C.

1.1 Random community matrices

We first discuss the construction of random community matrices: (i) Each species is randomly assigned to $\mathcal{K}$ sets. We denote that $g_i = 1$ if species i belongs to set $g, g = 1, 2, \cdots, \mathcal{G}$. (ii) For different species i and j, if $g_i g_j = 1$, we draw a random value p from a uniform distribution $U[0, 1]$. (iii) If $p \leq C$, we sample M_{ij}^g and M_{ji}^g from a normal distribution $N(0, \sigma^2)$, respectively. Otherwise $M_{ij}^g = M_{ji}^g = 0$, $g = 1, 2, \cdots, \mathcal{G}$. (iv) For the community matrix $\boldsymbol{M}$, we have $M_{ij} = \sum_{g=1}^{\mathcal{G}} M_{ij}^g$ if $i \neq j$ and we set diagonal terms $M_{ii} = -d$ ($d > 0$), $i = 1, 2, \cdots, S$.

For the corresponding random community matrices in unstructured systems, we construct them following the classical way shown in [1].

1.2 Exploitative community matrices

For exploitative community matrices, step (i) is identical to constructing random community matrices in S1.1. Subsequent steps are as follows: (ii) If $g_i g_j = 1$, for pair of interactions $\left(M_{ij}^g, M_{ji}^g\right)_{i>j}$, we draw a random value p_1 from a uniform distribution $U[0,1]$. (iii) If $p_1 \leq C$, we draw another random value p_2 from a uniform distribution $U[0,1]$. (iv) if $p_2 < 0.5$, we draw M_{ij}^g from $|N\left(0,\sigma^2\right)|$ and M_{ji}^g from $-|N\left(0,\sigma^2\right)|$. Otherwise, we do the opposite. (v) if $p_1 > C$, M_{ij}^g and M_{ji}^g are set to be 0, $g = 1, 2, \cdots, \mathcal{G}$. (vi) For the community matrix $\boldsymbol{M}$, we have $M_{ij} = \sum_{g=1}^{\mathcal{G}} M_{ij}^g$ if $i \neq j$ and we set diagonal terms $M_{ii} = -d$ $(d > 0)$, $i = 1, 2, \cdots, S$.

For the corresponding exploitative community matrices in unstructured systems, we construct them following the classical way shown in [1].

1.3 Mutualistic community matrices

For mutualistic community matrices, step (i) is identical to constructing random community matrices in S1.1. Subsequent steps are as follows: (ii) If $g_i g_j = 1$, for pair of interactions $(M_{ij}^g, M_{ji}^g)_{i>j}$, we draw a random value p from a uniform distribution $U[0,1]$. (iii) If $p \leq C$, M_{ij}^g and M_{ji}^g are drawn from a half-normal distribution $|N(0,\sigma^2)|$. Otherwise both M_{ij}^g and M_{ji}^g are 0, $g = 1, 2, \cdots, \mathcal{G}$. (iv) For the community matrix $\boldsymbol{M}$, we have $M_{ij} = \sum_{g=1}^{\mathcal{G}} M_{ij}^g$ if $i \neq j$ and we set diagonal terms $M_{ii} = -d$ $(d > 0)$, $i = 1, 2, \cdots, S$.

For the corresponding mutualistic community matrices in unstructured systems, we construct them following the classical way shown in [1].

1.4 Competitive community matrices

For competitive community matrices, steps (i), (ii), and (iv) are identical to constructing mutualistic community matrices in S1.3. And we have: (iii) If $p \leq C$, M_{ij}^g and M_{ji}^g are drawn from a negative half-normal distribution $-|N\left(0,\sigma^2\right)|$. Otherwise both M_{ij}^g and M_{ji}^g are 0, $g = 1, 2, \cdots, \mathcal{G}$.

For the corresponding competitive community matrices in unstructured systems, we construct them following the classical way shown in [1].

2 Stability estimation for different interaction types

Here we derive the stability criteria for different interaction types (random, exploitative, mutualistic, and competitive) in set structured systems. While our estimation of stability criteria in set structured

systems is approximate, it offers valuable insights into the general trends of stability. Precisely quantifying the stability of such systems can be challenging, but our theoretical framework adeptly captures the underlying dynamics, enabling us to predict the stability of set structured systems.

2.1 Expected number of common sets for two species

We begin with calculating the expected number of common sets, $\mathcal{H}$, for two randomly chosen, distinct species [2]. For any species i and j, the number of sets, $\mathcal{H}_{ij}$, that they have in common is

$$\mathcal{H}_{ij} = \sum_{g=1}^{\mathcal{G}} g_i g_j.$$

Then we have

$$\mathcal{H} = \langle \mathcal{H}_{ij} \rangle = \left\langle \sum_{g=1}^{\mathcal{G}} g_i g_j \right\rangle.$$

Since each species belongs to $\mathcal{K}$ sets, the possible number of sets two species have in common is $k \in \{0, 1, \cdots, \mathcal{K}\}$. The probability that $\mathcal{H}_{ij} = k$ can be computed as

$$P\left(\mathcal{H}_{ij} = k\right) = \begin{pmatrix} \mathcal{K} \\ k \end{pmatrix} \frac{\begin{pmatrix} \mathcal{G} - \mathcal{K} \\ \mathcal{K} - k \end{pmatrix}}{\begin{pmatrix} \mathcal{G} \\ \mathcal{K} \end{pmatrix}}.$$

To determine the specific form of $\mathcal{H}$, we sum up the product of k and the corresponding probability for all possible values of k:

$$\mathcal{H} = \sum_{k=0}^{\mathcal{K}} k P\left(\mathcal{H}_{ij} = k\right) = \sum_{k=0}^{\mathcal{K}} k \begin{pmatrix} \mathcal{K} \\ k \end{pmatrix} \frac{\begin{pmatrix} \mathcal{G} - \mathcal{K} \\ \mathcal{K} - k \end{pmatrix}}{\begin{pmatrix} \mathcal{G} \\ \mathcal{K} \end{pmatrix}}. \tag{S1}$$

Simplifying Eq. (S1), we have

$$\mathcal{H} = \frac{\mathcal{K}^2}{\mathcal{G}}. \tag{S2}$$

According to Eq. (S2), we obtain that the expected number of common sets for any two species

is given by $\frac{\mathcal{K}^2}{\mathcal{G}}$. Moreover, we calculate the expected interaction times, T, for any two species

$$T = C\mathcal{H} = C\frac{\mathcal{K}^2}{\mathcal{G}}.$$

Next, we derive the corresponding stability criteria for different interaction types in set structured systems.

2.2 Random

For random communities in set structured systems, we set the diagonal terms of $\boldsymbol{M}$ to be 0 in the initial. Following our discussion in S1.1 and S2.1, for any two species i and j $(i \neq j)$, we have

$$\begin{cases} \mathbb{E}\left(M_{ij}\right)_{i\neq j} = T\mathbb{E}\left(X\right) \\ \\ \mathrm{Var}\left(M_{ij}\right)_{i\neq j} = T\mathrm{Var}\left(X\right) \end{cases},$$

where $X \sim N\left(0, \sigma^2\right)$. Consequently, we obtain

$$\begin{cases} \mathbb{E}\left(M_{ij}\right)_{i\neq j} = 0 \\ \\ \mathrm{Var}\left(M_{ij}\right)_{i\neq j} = C\frac{\mathcal{K}^2}{\mathcal{G}}\sigma^2 \end{cases}.$$

We further estimate the largest real part of the eigenvalues of $\boldsymbol{M}$. According to circular law [1, 3], the eigenvalue distribution of $\boldsymbol{M}/(\sigma\sqrt{SC\frac{\mathcal{K}^2}{\mathcal{G}}})$ satisfies that: for any eigenvalue λ, as $S \to \infty$, we have

$$\|\lambda\| \leq 1. \tag{S3}$$

According to Eq. (S3), the largest real part of the eigenvalues of $\boldsymbol{M}$ approximates to $\sigma\sqrt{SC\frac{\mathcal{K}^2}{\mathcal{G}}}$ when S is sufficiently large.

Next, we take the diagonal strength into account. As shown in S1.1, we set $M_{ii} = -d$ for any species i, and the largest real part of the eigenvalues of $\boldsymbol{M}$ approximates to $\sigma\sqrt{SC\frac{\mathcal{K}^2}{\mathcal{G}}} - d$ as $S \to \infty$. Then we get the stability criterion for random cases

$$\sigma\sqrt{SC\frac{\mathcal{K}^2}{\mathcal{G}}} < d,$$

where $\mathcal{G}$ is the number of sets and $\mathcal{K}$ is the number of sets each species belongs to.

Compared with the stability criterion for unstructured systems in the random case

$$\sigma\sqrt{SC} < d.$$

We find that the set structure stabilizes the networked system if

$$\sigma\sqrt{SC\frac{\mathcal{K}^2}{\mathcal{G}}} < \sigma\sqrt{SC},$$

which equals to

$$\mathcal{G} > \mathcal{K}^2.$$

2.3 Exploitative

For exploitative communities, we also set the diagonal terms of $\boldsymbol{M}$ to be 0 first. Following our discussion in S1.3 and S2.1, for any two species i and j $(i \neq j)$, we have

$$\begin{cases} \mathbb{E}\left(M_{ij}\right)_{i\neq j} = 0 \\ \\ \mathbb{E}\left(M_{ij}M_{ji}\right)_{i\neq j} = -C\frac{\mathcal{K}^2}{\mathcal{G}}\mathbb{E}^2\left(|X|\right) \\ \\ \mathrm{Var}\left(M_{ij}\right)_{i\neq j} = C\frac{\mathcal{K}^2}{\mathcal{G}}\mathrm{Var}\left(X\right) \end{cases},$$

where $X \sim N\left(0, \sigma^2\right)$. Consequently, we obtain

$$\begin{cases} \mathbb{E}\left(M_{ij}\right)_{i\neq j} = 0 \\ \\ \mathbb{E}\left(M_{ij}M_{ji}\right)_{i\neq j} = -C\frac{\mathcal{K}^2}{\mathcal{G}}\frac{2}{\pi}\sigma^2 \\ \\ \mathrm{Var}\left(M_{ij}\right)_{i\neq j} = C\frac{\mathcal{K}^2}{\mathcal{G}}\sigma^2 \end{cases}. \tag{S4}$$

Here we consider the following theorem [4]: for a matrix $\boldsymbol{A}$, if its elements A_{ij} satisfy that

$$\begin{cases} \mathbb{E}\left(A_{ij}\right)=0 \\ \\ \mathbb{E}\left(A_{ij}A_{ji}\right)=\tau/S \\ \\ \operatorname{Var}\left(A_{ij}\right)=1/S \end{cases}, \tag{S5}$$

then the eigenvalues of $\boldsymbol{A}$ are uniformly distributed on an ellipse if S is sufficiently large

$$(x/a)^2+(y/b)^2 \leq 1, \tag{S6}$$

where $a=1-\tau$ and $b=1+\tau$.

According to Eqs. (S4), (S5), the eigenvalues of $\boldsymbol{M}/(\sigma\sqrt{SC\frac{\mathcal{K}^2}{\mathcal{G}}})$ are distributed on the ellipse shown in Eq. (S6), where

$$\tau=\frac{S\mathbb{E}\left(M_{ij}M_{ji}\right)_{i\neq j}}{SC\frac{\mathcal{K}^2}{\mathcal{G}}\sigma^2}=-\frac{2}{\pi},$$

thus the largest real part of the eigenvalues of $\boldsymbol{M}$ approximates to $\sigma\sqrt{SC\frac{\mathcal{K}^2}{\mathcal{G}}}\,(1-2/\pi)$ as $S\to\infty$.

Taking the diagonal strength into account, we have that the largest real part of the eigenvalues of $\boldsymbol{M}$ approximates to $\sigma\sqrt{SC\frac{\mathcal{K}^2}{\mathcal{G}}}\,(1-2/\pi)-d$ as $S\to\infty$.

Then we get the stability criterion for exploitative cases

$$\sigma\sqrt{SC\frac{\mathcal{K}^2}{\mathcal{G}}}\,(1-2/\pi)<d.$$

Similar to the random case, the set structure stabilizes the networked system in the exploitative community if

$$\mathcal{G}>\mathcal{K}^2.$$

2.4 Mutualistic

For mutualistic communities, we follow our discussion in S1.2 and S2.1 and have

$$\begin{cases} \mathbb{E}\left(M_{ij}\right)_{i\neq j}=C\frac{\mathcal{K}^2}{\mathcal{G}}\mathbb{E}\left(|X|\right) \\ \\ \operatorname{Var}\left(M_{ij}\right)_{i\neq j}=C\frac{\mathcal{K}^2}{\mathcal{G}}\mathbb{E}\left(X^2\right)-\left(C\frac{\mathcal{K}^2}{\mathcal{G}}\mathbb{E}\left(|X|\right)\right)^2 \end{cases},$$

where $X \sim N\left(0, \sigma^2\right)$. Consequently, we obtain

$$\begin{cases} \mathbb{E}\left(M_{ij}\right)_{i\neq j} = C\frac{\mathcal{K}^2}{\mathcal{G}}\sigma\sqrt{\frac{2}{\pi}} \\ \\ \mathrm{Var}\left(M_{ij}\right)_{i\neq j} = C\frac{\mathcal{K}^2}{\mathcal{G}}\sigma^2 - C^2\frac{\mathcal{K}^4}{\mathcal{G}^2}\sigma^2\frac{2}{\pi} \end{cases}.$$

With increasing S, the row sum of $\boldsymbol{M}$ approximates to

$$\sum_{j=1}^{S} M_{ij} \approx -d + (S-1)\, C\frac{\mathcal{K}^2}{\mathcal{G}}\sigma\sqrt{\frac{2}{\pi}}.$$

According to Gershgorin circle theorem [3, 5], for sufficiently large S, the largest real part of the eigenvalues of $\boldsymbol{M}$ is $-d + (S-1)\, C\frac{\mathcal{K}^2}{\mathcal{G}}\sqrt{\frac{2}{\pi}}$.

Next, we get the stability criterion for mutualistic cases

$$(S-1)\, C\frac{\mathcal{K}^2}{\mathcal{G}}\sigma\sqrt{\frac{2}{\pi}} < d.$$

Compared with the stability criterion for unstructured systems in mutualistic cases

$$(S-1)\, C\sigma\sqrt{\frac{2}{\pi}} < d,$$

we obtain that the set structure stabilizes the networked system if

$$\mathcal{G} > \mathcal{K}^2.$$

2.5 Competitive

For competitive interactions, we have

$$\begin{cases} \mathbb{E}\left(M_{ij}\right)_{i\neq j} = -C\frac{\mathcal{K}^2}{\mathcal{G}}\mathbb{E}\left(|X|\right) \\ \\ \mathbb{E}\left(M_{ij}M_{ji}\right)_{i\neq j} = C\frac{\mathcal{K}^2}{\mathcal{G}}\mathbb{E}^2\left(|X|\right) \\ \\ \mathrm{Var}\left(M_{ij}\right)_{i\neq j} = C\frac{\mathcal{K}^2}{\mathcal{G}}\mathbb{E}\left(X^2\right) - \left(C\frac{\mathcal{K}^2}{\mathcal{G}}\mathbb{E}\left(|X|\right)\right)^2 \end{cases}, \tag{S7}$$

where $X \sim N\left(0, \sigma^2\right)$. Consequently, we obtain

$$\begin{cases} \mathbb{E}\left(M_{ij}\right)_{i \neq j} = -C\frac{\mathcal{K}^2}{\mathcal{G}}\sigma\sqrt{\frac{2}{\pi}} \\ \\ \mathbb{E}\left(M_{ij}M_{ji}\right)_{i \neq j} = C\frac{\mathcal{K}^2}{\mathcal{G}}\frac{2}{\pi}\sigma^2 \\ \\ \mathrm{Var}\left(M_{ij}\right)_{i \neq j} = C\frac{\mathcal{K}^2}{\mathcal{G}}\sigma^2 - C^2\frac{\mathcal{K}^4}{\mathcal{G}^2}\sigma^2\frac{2}{\pi} \end{cases}. \tag{S8}$$

According to Allesina and Tang [1], we first consider the eigenvalue distribution of the matrix $\boldsymbol{N}$, which is defined as

$$\boldsymbol{N} = \boldsymbol{M} + (d + a)\,\boldsymbol{I} - a \cdot \mathbf{1} \cdot \mathbf{1}^{\mathrm{T}}, \tag{S9}$$

where $a = -C\frac{\mathcal{K}^2}{\mathcal{G}}\mathbb{E}\left(|X|\right)$.

Combining Eqs. (S7)-(S9), we have

$$\begin{cases} \mathbb{E}\left(N_{ij}\right)_{i \neq j} = 0 \\ \\ \mathbb{E}\left(N_{ij}N_{ji}\right)_{i \neq j} = C\frac{\mathcal{K}^2}{\mathcal{G}}\left(1 - C\frac{\mathcal{K}^2}{\mathcal{G}}\right)\frac{2}{\pi}\sigma^2 \\ \\ \mathrm{Var}\left(N_{ij}\right)_{i \neq j} = C\frac{\mathcal{K}^2}{\mathcal{G}}\sigma^2 - C^2\frac{\mathcal{K}^4}{\mathcal{G}^2}\sigma^2\frac{2}{\pi} \end{cases}. \tag{S10}$$

Furthermore, according the theorem in Eqs. (S4)-(S6), $\boldsymbol{N}$ is now an elliptic matrix. Thus, the largest real part of the eigenvalues of $\boldsymbol{N}$ can be approximated as

$$\sqrt{S\mathrm{Var}\left(N_{ij}\right)_{i \neq j}}\left(1 + \mathbb{E}\left(N_{ij}N_{ji}\right)_{i \neq j}/\mathrm{Var}\left(N_{ij}\right)_{i \neq j}\right),$$

when S is sufficiently large [1].

Then, according to Allesina and Tang [1], we obtain the largest real part of the eigenvalues of $\boldsymbol{M}$, λ_1, which approximates to

$$\lambda_1 = \sqrt{S\mathrm{Var}\left(N_{ij}\right)_{i \neq j}}\left(1 + \mathbb{E}\left(N_{ij}N_{ji}\right)_{i \neq j}/\mathrm{Var}\left(N_{ij}\right)_{i \neq j}\right) + C\frac{\mathcal{K}^2}{\mathcal{G}}\mathbb{E}\left(|X|\right) - d, \tag{S11}$$

when S is sufficiently large.

According to Eqs. (S10), (S11), we get the stability criterion for competitive cases

$$\sigma\left(F(C\frac{\mathcal{K}^2}{\mathcal{G}})+C\frac{\mathcal{K}^2}{\mathcal{G}}\sqrt{2/\pi}\right)<d,$$

where $F(x)$ satisfies that

$$F(x)=\sqrt{Sx\left(1-2\frac{x}{\pi}\right)}\left(1+\frac{2\,(1-x)}{\pi-2x}\right).$$

Since the stability of a competitive networked system is non-linearly related to the connectivity C, there is no mathematical condition in which the set structure can improve community stability.

3 Validation on simulated data

We undertake an extensive series of simulated experiments to validate the tenets of our theoretical analysis in the main text. Here we provide a concise exposition of our simulated experimentation approach in this section. Principally, we employ Monte Carlo simulations as the primary tool for validating our theoretical predictions of the critical $\mathcal{K}^2/\mathcal{G}$ to stabilize set structured systems. Specifically, we conduct a significant number of independent Monte Carlo simulations in each experiment, and calculate the average statistical outcomes derived from these simulations. Subsequently, we perform a comparative analysis between the statistical data and our theoretical predictions. These simulations conclusively demonstrate the fidelity of our theoretical analysis in accurately predicting the critical $\mathcal{K}^2/\mathcal{G}$ in set structured systems (Figs. 2-6).

Additionally, we systematically generate a substantial volume of data by utilizing Monte Carlo simulations. Subsequently, we subject these datasets to a comprehensive analysis within the framework of our model. By employing our model on these datasets, we establish precise quantitative distinctions compared to well-established benchmarks, specifically unstructured systems. The examination of these simulated datasets distinctly highlights the conspicuous stabilizing influence of set structures on community stability when $\mathcal{K}^2/\mathcal{G}<1$ (Fig. 4).

Supplementary References

[1] Allesina S and Tang S. Stability criteria for complex ecosystems. *Nature* 2012; **483**: 205–208.

[2] Tarnita CE, Antal T, Ohtsuki H *et al.* Evolutionary dynamics in set structured populations. *Proc. Natl. Acad. Sci.* 2009; **106**: 8601–8604.

[3] Girko VL. Circular law. *Theory Probab. Its Appl.* 1985; **29**: 694–706.

[4] Sommers HJ, Crisanti A, Sompolinsky H *et al.* Spectrum of large random asymmetric matrices. *Phys. Rev. Lett.* 1988; **60**: 1895–1898.

[5] Tao T, Vu V and Krishnapur M. Random matrices: universality of ESDs and the circular law. *Ann. Probab.* 2010; **38**: 2023–2065.

Supplementary Figures

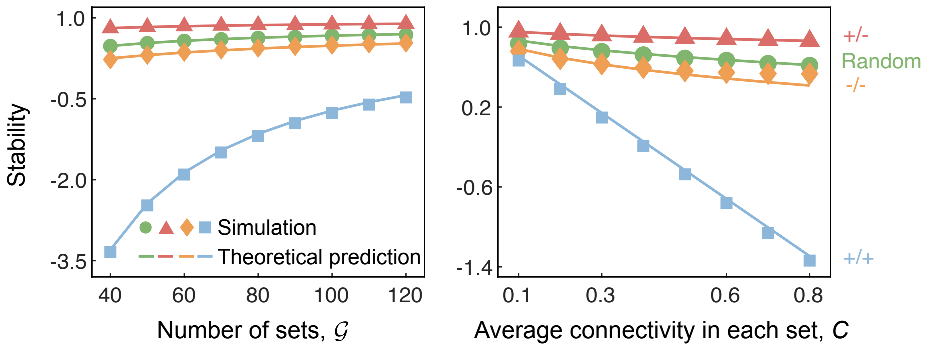

Supplemental Figure S1: Theoretical predictions for the stability of set structured systems. We show the effect of the number of sets $\mathcal{G}$ and average connectivity C on the stability of set structured systems with different interaction types (random, exploitative, mutualistic, and competitive) through both theoretical analysis (lines) and numerical simulations (dots). (a) We set the number of species $S = 400$, the number of sets each species belongs to $\mathcal{K} = 6$, average connectivity in each set $C = 0.3$, and vary the number of sets $\mathcal{G}$ from 40 to 120. (b) We set the number of species $S = 400$, the number of sets each species belongs to $\mathcal{K} = 6$, the number of sets $\mathcal{G} = 200$, and vary average connectivity in each set C from 0.1 to 0.8. Each simulation result is obtained from over 50 replicates.

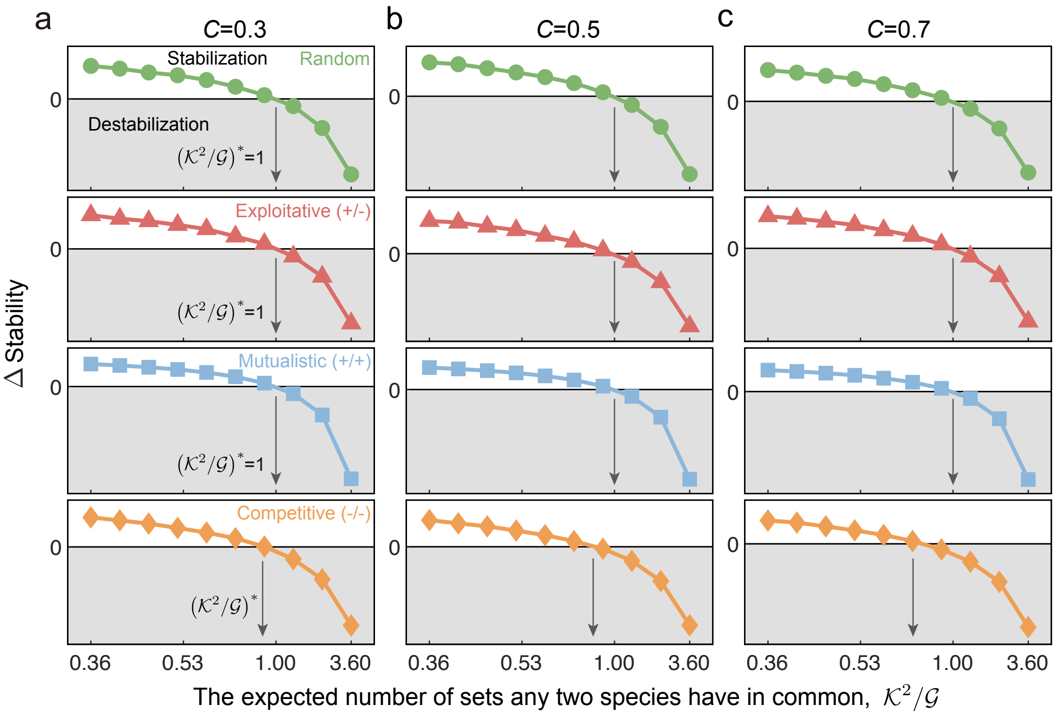

Supplemental Figure S2: Confirmation of the simple rule, $\mathcal{K}^2/\mathcal{G} < 1$, in set structured systems with various average connectivity C. (a-c) The ΔStability of set structured and corresponding unstructured systems with increasing $\mathcal{K}^2/\mathcal{G}$ in dense ($C = 0.3$), middle ($C = 0.5$), and sparse ($C = 0.7$) communities with different interaction types, respectively. We set the number of species $S = 500$. Other parameters are the same as those in Fig. 3.

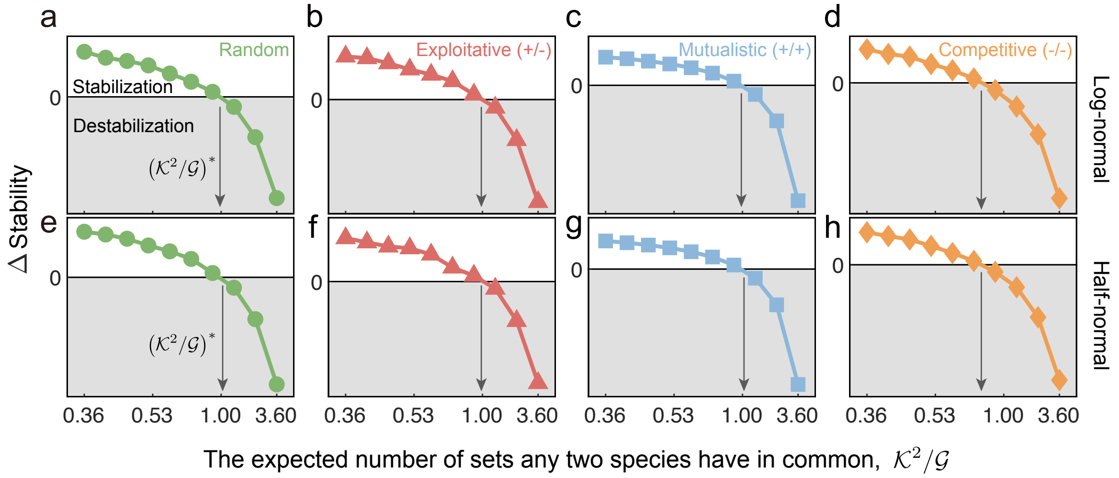

Supplemental Figure S3: Confirmation of the simple rule with diverse equilibrium abundances. The ΔStability of set structured and corresponding unstructured systems with diverse equilibrium abundance (log-normal and half-normal). (a-d) We sample $X_i{}^*$ from the log-normal distribution with log-mean 0 and log-standard deviation 0.05. (e-h) We sample $X_i{}^*$ from the half-normal distribution $|X|$, where $X \sim N(1, \sigma^2)$ and $\sigma = 0.05$. Other parameters are the same as those in Fig. 6e-h.

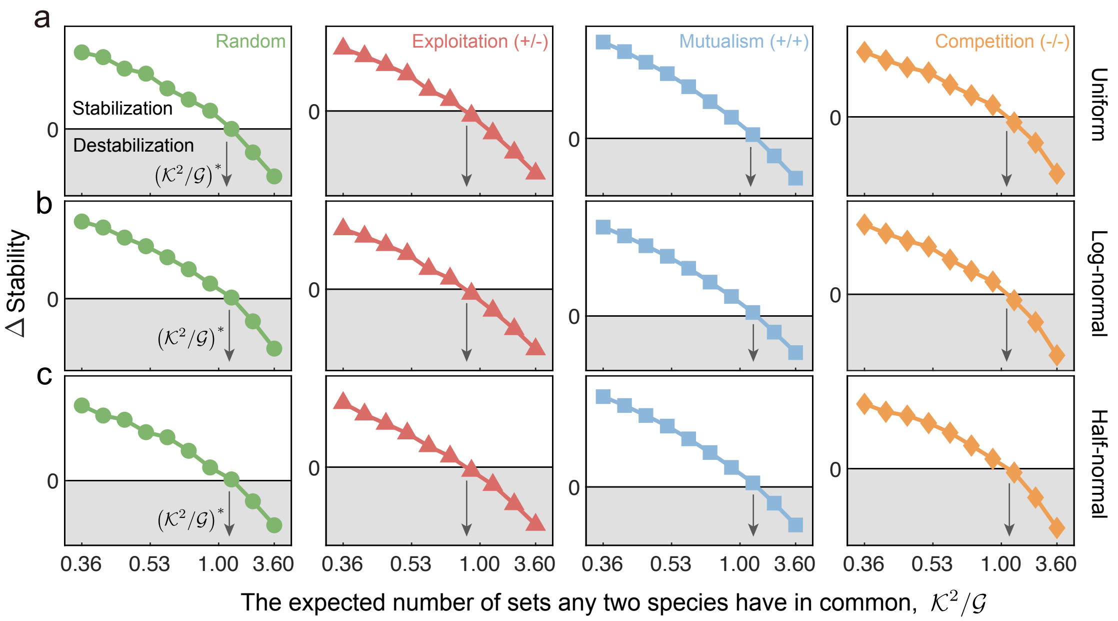

Supplemental Figure S4: Confirmation of the simple rule with diverse equilibrium abundances in heterogeneous set structured systems. The ΔStability of set structured and corresponding unstructured systems with diverse equilibrium abundance distributions (uniform, log-normal and half-normal) in heterogeneous communities. (a) We sample ${X_i}^*$ from the uniform distribution on $[0.95, 1.05]$. (b) We sample ${X_i}^*$ from the log-normal distribution with log-mean 0 and log-standard deviation 0.02. (c) We sample ${X_i}^*$ from the half-normal distribution $|X|$, where $X \sim N(1, \sigma^2)$ and $\sigma = 0.02$. We set the average connectivity $C = 0.2$. Other parameters are the same as those in Fig. 6e-h.

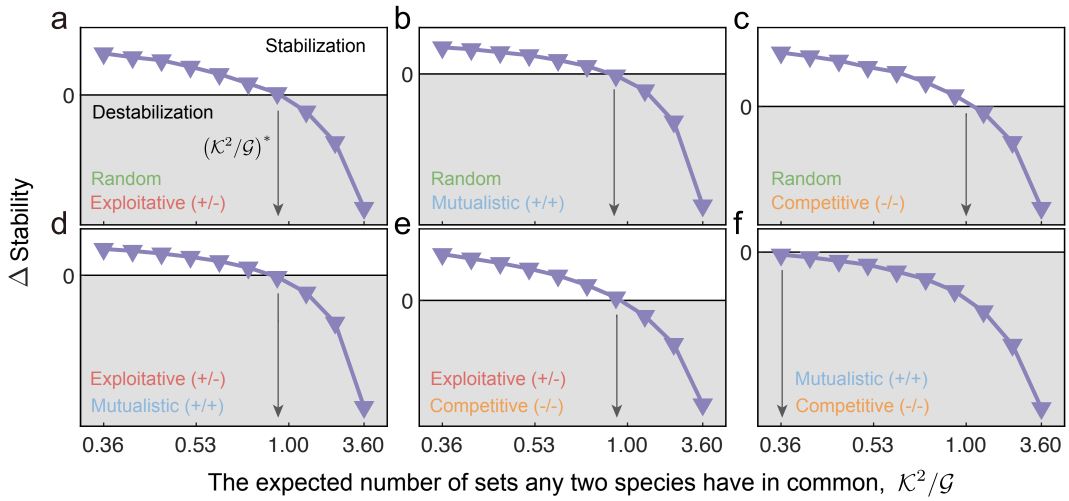

Supplemental Figure S5: ΔStability of set structured and corresponding unstructured systems with mixed interaction types. Interaction types in each set are randomly chosen from random, exploitative, mutualistic, and competitive interactions. (a-f) ΔStability of six different random compositions involving two interaction types. Other parameters are the same as those in Fig. 6e-h.

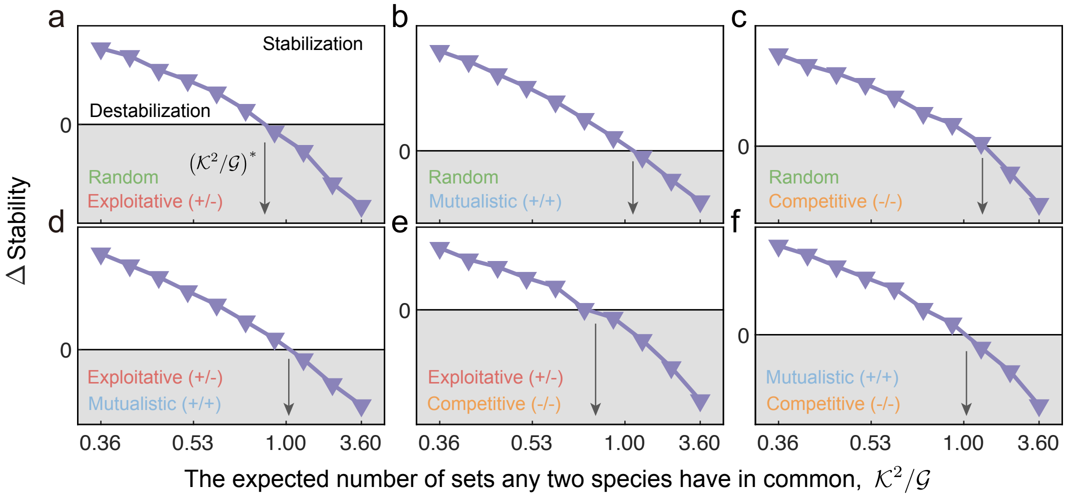

Supplemental Figure S6: ΔStability of heterogeneous set structured and corresponding unstructured systems with mixed interaction types. We set the average connectivity $C = 0.2$ in both heterogeneous set structured and unstructured systems. Other parameters are the same as those in Fig. S5.

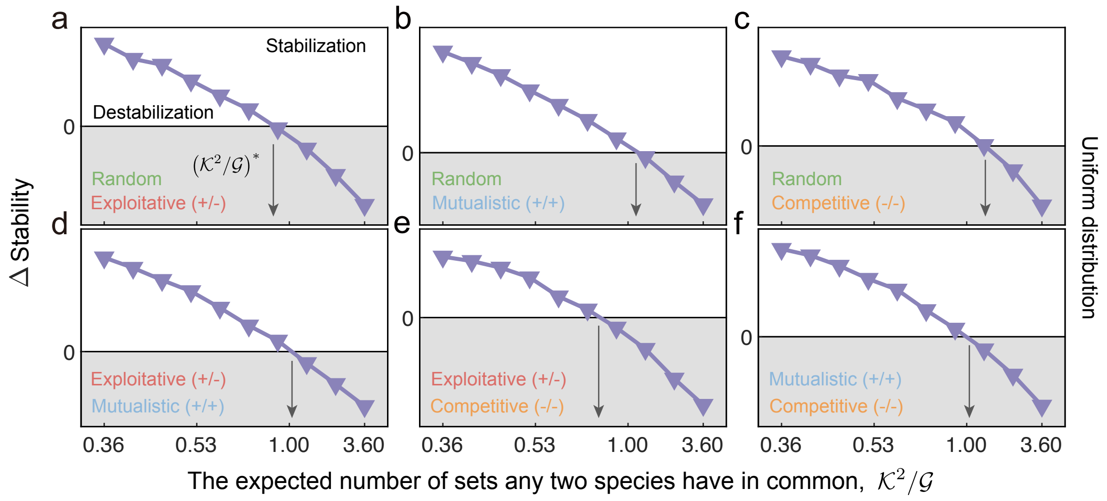

Supplemental Figure S7: ΔStability of heterogeneous set structured and corresponding unstructured systems with mixed interaction types under uniform distribution equilibrium abundance. We sample X_i^* from the uniform distribution on $[0.95, 1.05]$. Other parameters are the same as those in Fig. S6.

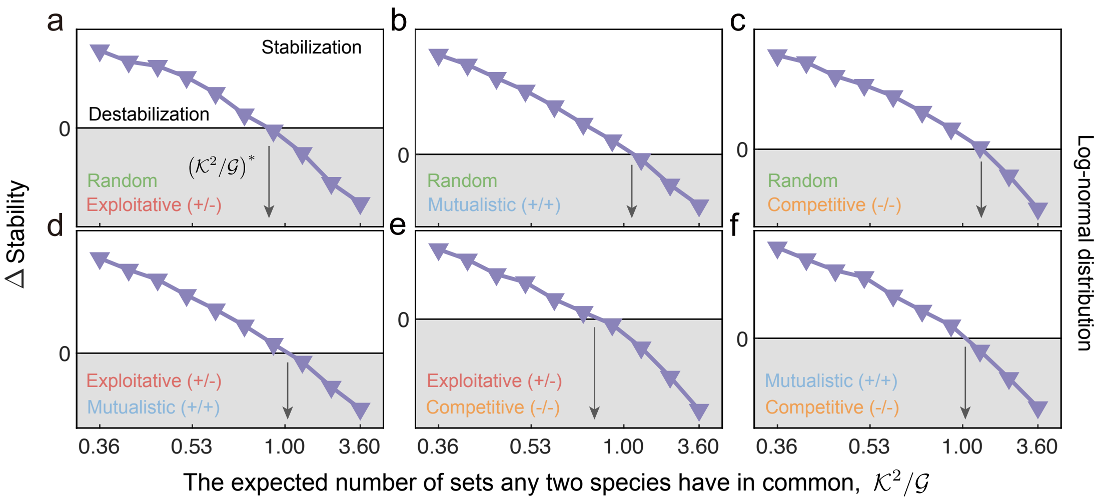

Supplemental Figure S8: ΔStability of heterogeneous set structured and corresponding unstructured systems with mixed interaction types under log-normal distribution equilibrium abundance. We sample $X_i{}^*$ from the log-normal distribution with log-mean 0 and log-standard deviation 0.02. Other parameters are the same as those in Fig. S6.

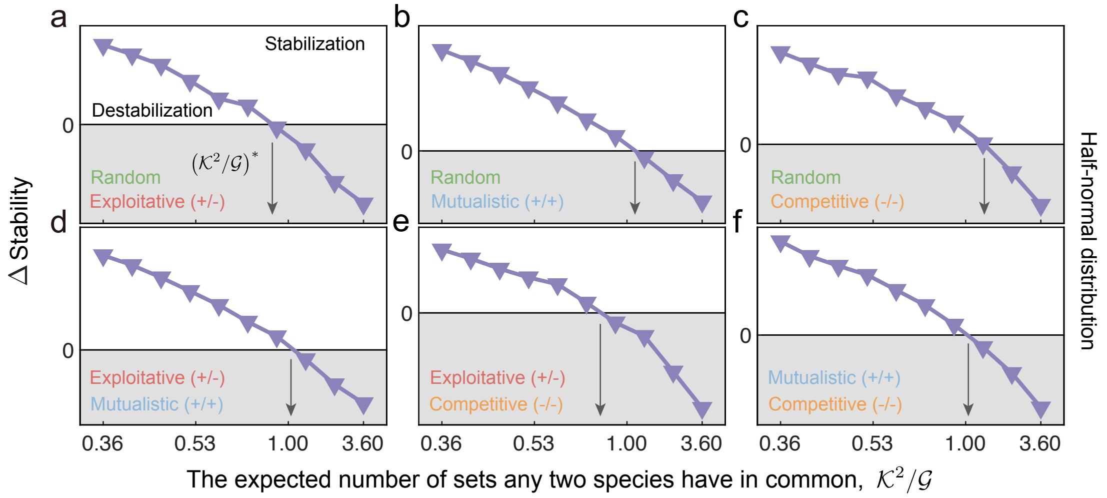

Supplemental Figure S9: ΔStability of heterogeneous set structured and corresponding unstructured systems with mixed interaction types under half-normal distribution equilibrium abundance. We sample ${X_i}^*$ from the half-normal distribution $|X|$, where $X \sim N(1, \sigma^2)$ and $\sigma = 0.02$. Other parameters are the same as those in Fig. S6.
